# XAI-Augmented Voting Ensemble Models for Heart Disease Prediction: A SHAP and LIME-Based Approach

**DOI:** 10.3390/bioengineering11101016

**Published:** 2024-10-12

**Authors:** Nermeen Gamal Rezk, Samah Alshathri, Amged Sayed, Ezz El-Din Hemdan, Heba El-Behery

**Affiliations:** 1 Department of Computer Science and Engineering, Faculty of Engineering, Kafrelsheikh University, Kafr_El_Sheikh 6860404, Egypt; nermeen_rezk@eng.kfs.edu.eg (N.G.R.); eng_heba_2010@eng.kfs.edu.eg (H.E.-B.); 2 Department of Information Technology, College of Computer and Information Sciences, Princess Nourah Bint Abdulrahman University, P.O. Box 84428, Riyadh 11671, Saudi Arabia; sealshathry@pnu.edu.sa; 3 Department of Electrical Energy Engineering, College of Engineering & Technology, Arab Academy for Science Technology & Maritime Transport, Smart Village Campus, Giza 12577, Egypt; 4 Industrial Electronics and Control Engineering Department, Faculty of Electronic Engineering, Menoufia University, Menoufia 32952, Egypt; 5 Department of Computer Science and Engineering, Faculty of Electronic Engineering, Menoufia University, Menoufia 32952, Egypt; ezzeldinhemdan@el-eng.menofia.edu.eg; 6 Structure and Materials Research Lab, Prince Sultan University, Riyadh 12435, Saudi Arabia

**Keywords:** explainable artificial intelligence (XAI), hybrid Ensemble learning, heart disease prediction, SHAP (SHapley Additive exPlanations), LIME (Local Interpretable Model-agnosticExplanations), voting algorithms

## Abstract

Ensemble Learning (EL) has been used for almost ten years to classify heart diseases, but it is still difficult to grasp how the “black boxes”, or non-interpretable models, behave inside. Predicting heart disease is crucial to healthcare, since it allows for prompt diagnosis and treatment of the patient’s true state. Nonetheless, it is still difficult to forecast illness with any degree of accuracy. In this study, we have suggested a framework for the prediction of heart disease based on Explainable artificial intelligence (XAI)-based hybrid Ensemble Learning (EL) models, such as LightBoost and XGBoost algorithms. The main goals are to build predictive models and apply SHAP (SHapley Additive expPlanations) and LIME (Local Interpretable Model-agnostic Explanations) analysis to improve the interpretability of the models. We carefully construct our systems and test different hybrid ensemble learning algorithms to determine which model is best for heart disease prediction (HDP). The approach promotes interpretability and transparency when examining these widespread health issues. By combining hybrid Ensemble learning models with XAI, the important factors and risk signals that underpin the co-occurrence of heart disease are made visible. The accuracy, precision, and recall of such models were used to evaluate their efficacy. This study highlights how crucial it is for healthcare models to be transparent and recommends the inclusion of XAI to improve interpretability and medical decisionmaking.

## 1. Introduction

Although heart disease is a major cause of death, it is important to note that not everyone with heart disease will eventually pass away. Individuals with heart diseases can live satisfied, full lives long into old age if they receive prompt, accurate diagnosis and proper treatment, which will significantly improve their overall quality of life. More recently, effective methods, such as hybrid Ensemble Learning (HEL) classification models, have been developed; these have been essential in providing the precise diagnoses of heart disease. A new area of study, including the explanation of the models used for the categorization of heart diseases, has emerged because of the development of explainable machine learning [1] approaches in recent years. 

We have used a comprehensive set of data on heart disease to use these classification methods in our ongoing research. Ensuring the fairness and dependability of the models that have been designed is a major emphasis of our work. To accomplish this, we have combined explainable artificial intelligence (XAI) methods with hybrid Ensemble Learning, which is intended to determine which model is the most reliable. In-depth analyses of ensemble classification methods are conducted in our study, including LightBoost and XGBoost algorithms, with preprocessing techniques, such as feature scaling, that transform the feature values to a similar scale, ensuring that all features contribute equally to the model and then using HEL with XAI. The two most popular frameworks for explaining artificial intelligence models are SHAP (SHapley Additive explanations) [2] and LIME (Local Interpretable Model-agnostic Explanations) [3]. 

The outcomes of our study clearly demonstrate the exceptional performance of the boosting algorithms, XGBoost and LightBoost, with metrics that show great promise. According to our research, the XGBoost and LightBoost classifier models show noticeably greater coverage and smaller set sizes, demonstrating their applicability and fairness for use in the prediction of heart disease. This study classifies heart diseases along with their explanations using hybrid Ensemble Learning algorithms. We have documented the performance metrics both with and without explanation. XGBoost and LightBoost are the models that are being examined. The study’s focus is SHAP and LIME. Additionally, feature contributions and feature weights were considered for feature selection based on the explanations. The hybrid Ensemble Learning based on XGBoost and LightBoost algorithms were fed the characteristics that had been shortlisted for further categorization, and metrics were tracked. To verify that their values had changed, the accuracies of the models with and without explanations were compared.

The study’s contributions are as follows:

▪ Out of the Artificial Intelligence models deployed for classification, the hybrid Ensemble Learning based on LightBoost and XGBoost algorithms presented the best results concerning performance metrics.
▪ The results of the hybrid Ensemble Learning based on the LightBoost and XGBoost algorithms classifier have been explained by explainable models: SHAP and LIME.
▪ To detect heart failure with significantly fewer resources, this study finds a feature subset that helps forecast the condition.
▪ Using hybrid Ensemble Learning based on LightBoost and XGBoost algorithms, this framework can effectively serve as a trigger to link heart disease patients to detect the presence of disease early on.

The remainder of the paper is structured as follows. Section 2 describes the relevant work using the materials and methodology that are provided in Section 3. Section 4 presents a visualization of feature importance, and Section 5 presents the results analysis. The limitations of this study are provided in Section 6, while the comparative analysis and conclusion are presented in Section 7.

## 2. Related Work

This section lists various relevant explainable artificial intelligence studies and their applications in the categorization of heart diseases. Recently, explainable AI has permeated healthcare classification schemes, as seen in the early detection of heart disease [4]. The author in [5] focuses on developing an explainable AI model to predict CVDs over a period of ten years. The open CVD study dataset was used to develop multiple machine learning models, i.e., KNN, Logistic Regression, XGBoost, CatBoost, Random Forest, and Decision tree. The models’ performance was assessed based on F1-score, accuracy, AUC, precision, recall, sensitivity, specificity, and the confusion matrix metrices. From this study, it is observed that the XGBoost model performs better than the other models with an accuracy of 89%. The SHAP explainable technique was later applied to all the models to understand their prediction for breaking the black box nature of machine learning models. 

A thorough method for building a system that uses explainable AI (XAI) and machine learning techniques to create a dependable and comprehensible predictive model for coronary artery disease (CAD) is presented in [6]. The key objectives are to build predictive models and apply SHAP analysis to improve the interpretability of the models. The proposed model is constructed and tested using different machine learning algorithms to determine which model is best for CAD prediction. After the model is selected, SHAP analysis is used to clarify how various features affect the model’s predictions, which helps to provide a better understanding of the underlying mechanisms that govern CAD classification. The paper emphasizes how crucial interpretability and predictive accuracy are when making medical decisions. 

In [7], the authors present a strategy that combines the interpretability provided by Shapley additive explanations (SHAP) with the accuracy of Bayesian optimization for hyperparameter tuning and the robustness of ensemble learning algorithms. Extreme gradient boosting (XGBoost), random forest, and adaptive boosting (AdaBoost) are the ensemble classifiers that are taken into consideration. It gives 0.971 and 0.989 for specificity and 0.921 and 0.975 for sensitivity on the Cleveland dataset and the Framingham dataset, respectively. In [8], the authors present cardiovascular illnesses using various machine learning and neural network models. The authors employed explainable artificial intelligence model-agnostic techniques to interpret the prediction. Experimentation data show that the multi-level model of artificial neural network (ANN) produces the best accuracy compared with other models.

The authors in [9] present three major contributions. First, a grouping of instances is used to handle missing values. To select the best features, a dual filter-based feature selection is presented. Then, Grey Wolf optimization is used to optimize the machine learning models’ hyperparameters. Collectively, these efforts seek to fill in the gaps in the data, enhance feature selection, and adjust model parameters to increase the resilience and effectiveness of machine learning applications.

In [10], the hybrid AI-based model CardioRiskNet is presented in this work. Data preprocessing, feature selection and encoding, explainable AI (XAI) integration, active learning, attention mechanisms, risk prediction and prognosis, assessment and validation, and deployment and integration are the seven components that make up the proposed CardioRiskNet. Prior to any further processing, the patient data is cleaned, missing value management and normalization is applied, and feature extraction is carried out. Subsequently, the most illuminating characteristics are chosen, and the numerical representation of the category variables is achieved. In a unique way, CardioRiskNet uses active learning to choose useful samples iteratively, increasing the effectiveness of its learning process, and its attention mechanism dynamically concentrates on the pertinent features for accurate risk prediction. 

Furthermore, the incorporation of XAI enhances decision-making interpretability and transparency. A convolutional neural network (CNN) long short-term memory (LSTM) (CNN-LSTM) network can be used to create an explainable artificial intelligence (XAI) framework for PCG-based VHD diagnosis. To attain high diagnostic accuracy, XAI is used to provide interpretability for the model’s predictions. PCG signals are enhanced using data augmentation techniques. The pertinent features are extracted from the PCG signals using mel-spectrograms, as in [11]. For the prediction of heart disease, the authors in [12] suggest an effective explainable recursive feature elimination with extreme Gradient Boosting (ERFEX) framework. ERFEX uses explainable AI methods to pinpoint important characteristics and tackle problems with class disparity. Using the ERFEX framework, they constructed several machines learning methods, including SHapley Additive exPlanations (SHAP) and the Support Vector Machine-based Synthetic Minority Over-sampling Technique (SVMSMOTE) for handling imbalanced classes and explainable feature selection. XGBoost and Random Forest classifiers are used in the ERFEX framework. 

An explainable machine learning technique was used in [13] to estimate the risk of CVD. To diagnose CVD, four machine learning models were used: extreme Gradient Boost (XGB), Random Forest (RF), K-nearest neighbor (KNN), and Decision Tree (DT). The models’ predictions were supported by Shapley Additive Explanations (SHAP). To aid with CVD diagnosis, a user interface was created using these models and explanations. In [14], they are proposing a lightweight artificial neural network (ANN) model. Utilizing forty-three input features from the 2021 Behavioral Risk Factor Surveillance System (BRFSS) dataset, the model generates balanced results from significantly unbalanced large survey data, outperforming previous models. This ANN model’s effectiveness is ascribed to its robustness in determining the likelihood of a myocardial infarction (MI) and its streamlined architecture, which lowers processing demands. 

In [15], a machine learning system for the early detection of heart disease utilizing many of the featured selection techniques is presented. Three different approaches—chi-square, analysis of variance (ANOVA), and mutual information (MI)—were used in the feature selection process. The final feature sets that were chosen were designated as SF-1, SF-2, and SF-3, in that order. The optimal method and the feature subset with the best fit were then ascertained using ten distinct machine learning classifiers. These classifiers were designated as (A1, A2, …, A10) and included LR, Naive Bayes, SVM, voting, XGBoost, AdaBoost, bagging, DT, KNN, and RF. In Table 1, the key contribution, methodology, and the advantages and disadvantages are highlighted to provide a description and comparison of some of the previous work.

## 3. Materials and Methodology

**Figure 1:**
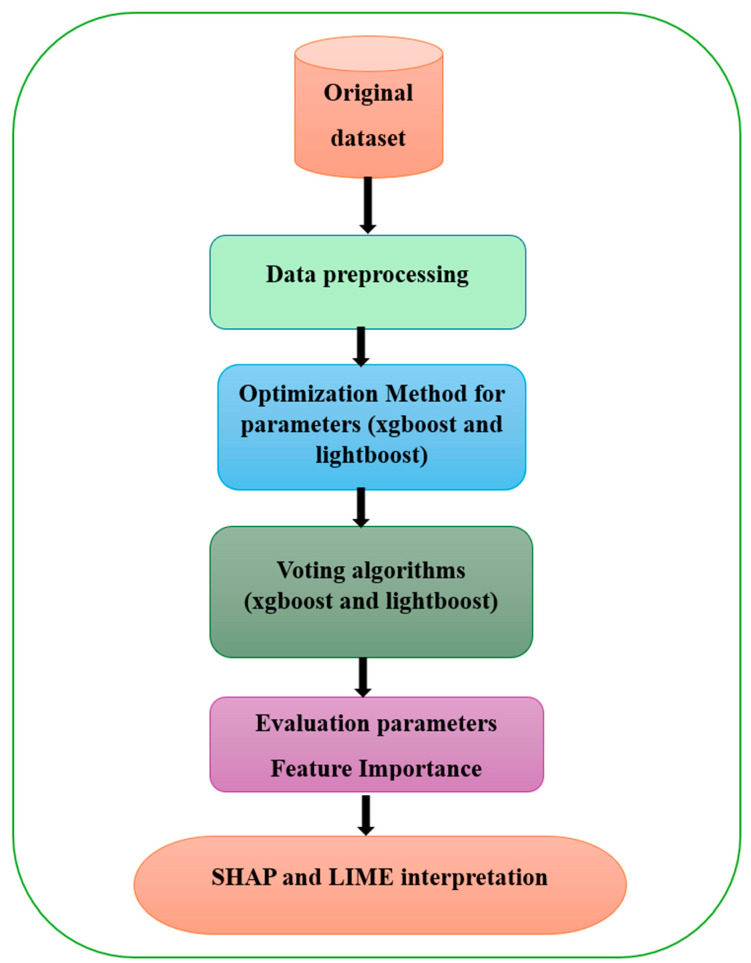
Framework for proposed system.

Our methodology for developing a system model involves several key steps: identifying the dataset, data preprocessing, identifying the optimization method for parameters (XGBoost and LightBoost), designing the model’s structure, training the model on the data, evaluating the model’s performance, and applying XAI techniques. This process is illustrated in Figure 1.

The framework shown in the figure illustrates the steps involved in the framework for heart disease prediction using hybrid models of ensemble learning, XGBoost, and LightGBM with XAI techniques such as SHAP and LIME. The detailed of each step as follows:

**(1) Original Dataset**: First, a raw dataset must be collected. The data would most likely include a variety of health-related factors that are typically considered when forecasting heart problems.

**(2) Data Preprocessing:** Before training the model, the input data must be preprocessed. The steps taken for data preparation are data cleaning, handling missing data, as well as other outlying values, variable transformation (normalization), and categorization, as well as data partitioning between training and test sets.

**(3) Optimization Method for Parameters (XGBoost and LightGBM)**: Thus, in this step, an optimization technique, such as Bayesian optimization, is exploited to tune the hyperparameter of XGBoost and LightGBM models to increase the accuracy. 

**(4) Voting Algorithms (XGBoost and LightGBM)**: The result of each of the two models (XGBoost and LightGBM) is then averaged, and a voting mechanism is used to help in reaching the final results. This Ensemble method aims at making a combination of the foregoing algorithms in such a way that the resulting accuracy is higher.

**(5) Evaluation Parameters and Feature Importance**: To assess the performance of the models, standard parameters, such as accuracy, precision, and recall, etc., are employed. Moreover, feature importance is computed to determine which features have the most significant effect in the classification of heart disease.

**(6) SHAP and LIME Interpretation**: Finally, explainable AI techniques, such as SHAP and LIME, are applied to interpret the model’s predictions. These techniques help to make the model’s decisions more transparent by showing how individual features contribute to specific predictions, allowing for better understanding for medical decision-making.

In summary, the proposed framework involves optimizing hybrid ensemble models, assessing their performance, and making the results interpretable using XAI methods.

### 3.1. Dataset

The dataset comprises 1190 patients with 11 predictive attributes, including demographics (age, sex), clinical measures (resting blood pressure, chest pain, cholesterol, heart rate, angina), and electrocardiographic data (ST segment, fasting blood sugar, resting ECG). The target variable indicates whether a patient has coronary heart disease (CHD), with 553 positive cases.

To create our predictive model, we analyzed a publicly available heart disease dataset from Kaggle [16]. This dataset contains 11 features, as outlined in Table 2. These features provide valuable information for understanding and predicting heart disease risk.

### 3.2. Methodology

One of the essential steps in preparing data for analysis and machine learning is data normalization. It involves converting the data into a uniform format to improve accuracy, minimize redundancy, and ensure consistency across all features. This process becomes especially critical when working with variables that have vastly different ranges, as it ensures that no feature disproportionately affects the analysis, provided there are no extreme outliers [17].

In [18], the authors employed a normalization technique known for its simplicity and effectiveness in supporting distance-based algorithms. It is fundamental to recognize that scaling can occasionally distort the original data, risking potential information loss. However, by utilizing normalization methods in our research, it effectively preserved the integrity and relationships between the original data values.

In machine learning, the Bayesian optimization in [19] is a potent method for hyperparameter optimization. It makes use of probabilistic models to effectively search the space and identify ideal parameter combinations. Bayesian optimization can intelligently balance exploration (testing novel configurations) and exploitation (focusing on promising regions) by building a surrogate model of the goal function. This strategy works especially well, because evaluating the objective function is costly, since it reduces the number of function evaluations needed to arrive at a good solution. Three main elements are usually involved in Bayesian optimization: an acquisition function (e.g., expected improvement, probability of improvement, entropy search) that directs the selection of the subsequent hyperparameter configuration to evaluate, a surrogate model (usually a Gaussian process or a random forest), and a prior distribution over the hyperparameters [20].

A common method for ensemble learning is voting algorithms [21], which aggregate the predictions of several base models to increase overall performance. Voting frequently produces better outcomes than any one model by combining the predictions of several models [22]. Two potent gradient boosting frameworks that are commonly utilized as foundation models in voting ensembles are XGBoost [23] and LightGBM [24]. Extreme gradient boosting, or XGBoost, is renowned for its precision and effectiveness. To avoid overfitting, it uses a gradient boosting architecture with regularization approaches. Big datasets and intricate models are no match for XGBoost.

In contrast, LightGBM is engineered for rapidity and effectiveness. To cut down on training time, it makes use of column-wise sampling and gradient boosting based on histograms. For real-time applications and large-scale datasets, LightGBM works especially well. We can take advantage of the complementing benefits of XGBoost and LightGBM by combining their capabilities in a voting ensemble to produce reliable and accurate predictions. We can take use of each model’s advantages and maybe outperform it when we use XGBoost and LightGBM together in a voting ensemble. By reducing overfitting, enhancing generalization, and boosting robustness against data noise, voting might be beneficial.

Understanding feature importance is essential to comprehending machine learning models’ internal mechanisms [25]. It assists in determining which features have the biggest impact on the predictions made by the model. Gaining an understanding of the underlying links between features and the goal variable, enhancing the interpretability of the model, and maybe streamlining it by eliminating superfluous features can all be facilitated by this information.

Explainable AI (XAI) techniques provide an effective way to determine the significance of features in model predictions [26]. Methods like such as SHAP (SHapley Additive exPlanations), LIME (Local Interpretable Model-agnostic Explanations), and permutation importance are commonly used to assess feature importance. These techniques will be applied to various model types, including decision trees, neural networks, and linear models, to evaluate the relative significance of distinct features influencing model outputs.

### 3.3. Performance Metrics

Let TP stand for true positive, TN for true negative, FP for false positive, and FN for false negative. Table 3 lists the many parameters that this article employed to assess our HDP approach [27].

## 4. Visualization of Feature Importance

An illustration of the several XAI methodology types, including model-specific and model-agnostic methods. It could show the connections and overlaps between several approaches as a Venn diagram or a hierarchical diagram. One may, for instance, incorporate LIME, saliency maps, counterfactual justifications, and feature importance. Within the SHAP framework, beeswarm is a potent visualization tool that offers insights into how various variables affect model predictions. The figure aids in our understanding of how various feature values affect the model’s output by displaying the spreading of SHAP values for each feature. The contribution of each characteristic to the forecast is indicated by its position on the x-axis, and each dot in the plot represents a data point. Further insight is provided by the fact that the color of the dot frequently matches the feature’s initial value.

Finding the features that most significantly affect the model’s predictions and comprehending how their effects change with various data points are two areas in which this visualization technique excels. Researchers can enhance the interpretability and reliability of their machine learning models and learn a great deal about the underlying mechanisms by utilizing SHAP plots with beeswarm. SHAP waterfall charts are very helpful in determining how different factors affect a given prediction. Each feature’s contribution to the forecast is represented by a bar, and the features are displayed in descending order of contribution to the prediction. Additionally, the figure displays the baseline forecast and the progressive contribution of every feature to the last prediction. It is simple to recognize the most crucial elements and comprehend how they work together to generate the desired result thanks to this visual portrayal.

## 5. Results and Analysis

**Figure 2:**
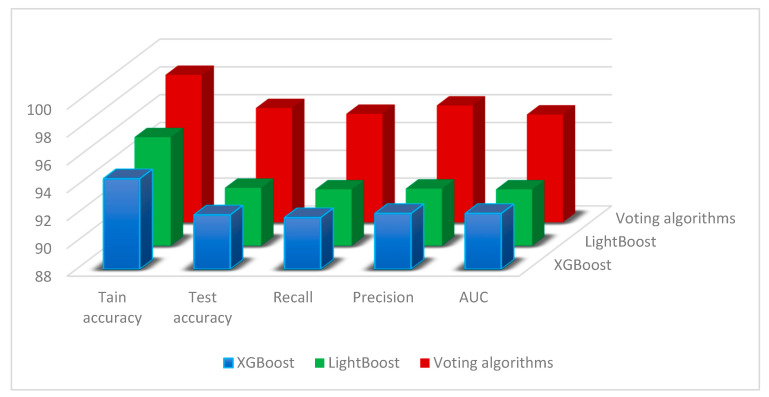
The comparison results of ensemble learning with voting technique.

We have gone to great lengths to present the outcomes and framework analysis in this section. A variety of performance metrics were employed in the algorithms’ evaluation. We also evaluated our model against other already used models, taking accuracy, recall, and precision into consideration, as shown in Table 4 and Figure 2.

In every case, our suggested voting system performed better in terms of accuracy, recall, and precision than the current techniques. We tested several algorithms’ performance on a wide range of datasets in our in-depth research. In comparison with baseline approaches and other state-of-the-art techniques, the findings repeatedly showed that our algorithm was able to successfully aggregate the votes from several classifiers, resulting in much better predictions, as shown in Figure 2.

Across all assessment metrics, the voting algorithm performed better than each of the separate base classifiers. This improvement might be ascribed to the ensemble method, which skillfully blends the advantages of several models. We used XAI approaches to comprehend the contributions of various features to the model’s predictions. According to the feature importance study, [list of top features] was crucial to the voting algorithm’s ability to make decisions. These results offer insightful information about the fundamental elements affecting the accuracy of the model and can be used to enhance subsequent iterations.

**Figure 3:**
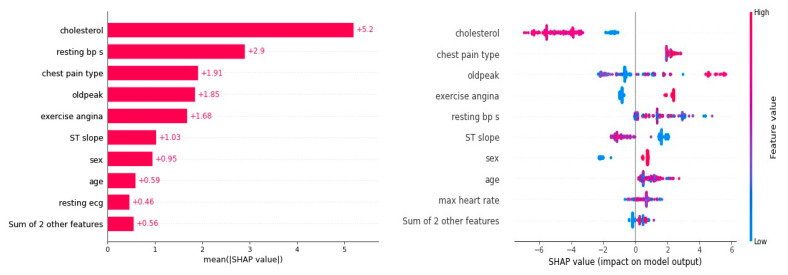
SHAP explanation.

The VEL model produced results that were better than those of other ML models, and the SHAP details were used to examine the main mechanism of these results. The VEL model’s average SHAP values, or feature significance, and the SHAP summary plot are shown in Figure 3. Cholesterol showed the highest mean SHAP value of (5.2), implying that it is the main factor that contributes effectively to heart disease prediction (HDP), based on the feature relevance in respect to the total model predictions. Resting BPs came in second on the list with a mean SHAP value of (2.9), while the effects on the model predictions were about similar for chest pain type (1.91), oldpeak (1.85), and exercise angina (1.68). ST slope (1.03), sex (0.95), age (0.59), and resting ECG (0.46) had the lowest impact.

The SHAP summary plot, as shown in Figure 3, shows the correlation between each feature value’s contribution and magnitude. According to VEL, the parameter cholesterol has the biggest effect on the HDP; lower cholesterol values resulted in lower HDP values, and vice versa. In summary, great heart disease prediction is indicated by a low HDP, which is a result of low cholesterol. Like cholesterol, resting BPs had a beneficial effect on the target, as did chest pain type. HDP falls when chest pain type and resting BPs are low, and vice versa. On the other hand, high HDP values correlated with high exercise angina levels, and vice versa. This suggests that since low exercise angina concentrations result in high HDP values, they are inappropriate for use in heart diagnosis.

**Figure 4:**
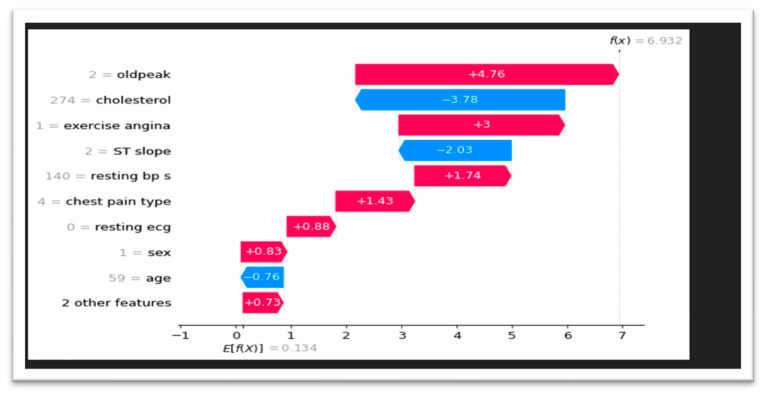
SHAP waterfall explanation.

We used SHAP waterfall charts to give a more detailed insight of feature contributions to model predictions. The relative significance of each factor influencing the outcome is shown by these visualizations, which break down each prediction into the sum of its component features. We were able to determine the major variables affecting the predictions of our model, such as cholesterol and oldpeak, by looking at these charts in Figure 4. Waterfall charts also made it possible to see feature interactions that would have gone unnoticed otherwise.

**Figure 5:**
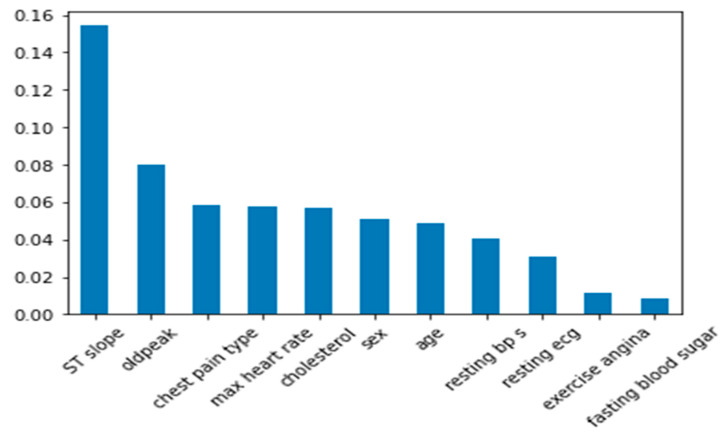
Feature importance using LIME explanation.

Especially in vital fields like healthcare, LIME is an effective tool for increasing the transparency and interpretability of complex machine learning models. Through the provision of insights into the parameters influencing model predictions, LIME can contribute to enhancing the efficacy and reliability of AI-driven heart disease prediction systems. Figure 5 shows the feature importance of datasets in predicting heart disease.

## 6. Limitations

From this study there are some potential limitations, as follows:

- **Generalization to diverse populations:** Although the Voting Ensemble model (VEL) achieved a 96.5% accuracy rate, the performance of the model may vary in real-world applications depending on the diversity of the population. The model may need to be retrained with more diverse data to improve generalization across different demographic groups and geographical regions.
- **Dependency on quality of input data:** The success of the VEL model relies heavily on the quality of genetic data, imaging results, and medical history. In cases where the input data are incomplete, inconsistent, or of poor quality, the accuracy of the model may decline.
- **Active learning limitations:** Although active learning enhances the VEL model by incorporating feedback from healthcare professionals, this requires continuous human intervention, which could become resource intensive. Additionally, the model may struggle with feedback delays or biased input from experts.
- **Complexity in clinical adoption:** Although the model takes XAI (explainable AI), active learning, and attention mechanism into account to enhance interpretability, the operator or the technician might have difficulties in understanding the model and using it because of all its complexities. To make sure that it is implemented effectively, it is crucial to provide good training and create designs that are easy to use.
- **Feature focus and risk factor exclusion:** The attention mechanism allows VEL to focus on certain patterns and features, but this could also lead to the exclusion of less obvious but still significant risk factors. It may overlook features not highlighted by its internal mechanisms, resulting in potentially biased or incomplete predictions.

These limitations highlight areas where the VEL model can be further enhanced for broader applicability in healthcare.

## 7. Conclusions

In conclusion, explainable heart disease may be predicted and prognosticated using the Voting Ensemble (VEL) model. The evaluation results of the model demonstrate its promise as a sophisticated and trustworthy model for assessing heart risk. The model achieved a 96.5% accuracy rate, which shows that it can perform better in real-world scenarios than the other approaches. VEL uses XAI, active learning, and an attention mechanism to examine genetic data, imaging results, and medical history. Healthcare practitioners can better grasp the risk assessment variables with the model’s clear and comprehensible risk forecasts. VEL performance can be enhanced by active learning, which incorporates feedback from healthcare professionals and selects informative examples. With the use of its attention mechanism, VEL can concentrate on pertinent patterns and features, picking up on minute but important risk prediction indicators. VEL robust functionality gives medical practitioners an effective tool for managing and predicting. AI’s potential for risk prediction and health monitoring is demonstrated by studies on stress detection utilizing physiological data and machine learning.

## Figures and Tables

**Table 1:** Comparative study of explainable AI (XAI) applications in heart disease detection.

Ref	Key Contribution	Methodology	Dataset	XAI Techniques	Advantages	Disadvantages
[4]	Systematic review of LIME and SHAP in Alzheimer’s disease detection	Literature review	Alzheimer’s disease	LIME, SHAP	Comprehensive overview of LIME and SHAP	Limited to Alzheimer’s disease
[5]	Application of XAI to heart disease prediction	Machine learning models (e.g., decision trees, random forests)	Heart disease	LIME, SHAP	Demonstrates the applicability of XAI in healthcare	Limited to a specific dataset
[7]	Optimized ensemble learning with XAI	Ensemble learning (e.g., random forest, gradient boosting)	Heart disease	LIME	Improved prediction accuracy through ensemble learning	May be complex to implement
[9]	Explainable AI model in heart disease classification using grey wolf optimization	Grey wolf optimization	Heart disease	LIME	Optimizes model performance through metaheuristic optimization	May be sensitive to parameter tuning
[10]	Hybrid AI-based model for explainable risk prediction and prognosis in cardiovascular disease	Hybrid AI model (e.g., deep learning, random forest)	Cardiovascular disease	LIME	Combines the strengths of different AI techniques	May be complex to interpret
[11]	Explainable AI for CNN-LSTM network in PCG-based valvular heart disease diagnosis	CNN-LSTM network	PCG recordings	LIME	Effective for analyzing time-series data	May require specialized knowledge for implementation
[14]	Identification of myocardial infarction probability using ANN with XAI	ANN	Imbalanced medical survey data	LIME, SHAP	Handles imbalanced data effectively	May be sensitive to hyperparameter tuning
[15]	Predicting heart diseases using machine learning and different data classification techniques	Machine learning (e.g., SVM, decision tree, random forest)	Heart disease	Not explicitly mentioned	Demonstrates the effectiveness of various machine learning algorithms	May not provide detailed explanations

**Table 2:** Feature descriptions.

SN	Attribute Name	Feature Type	Description
1	Age	Integer	Age in years
2	Sex	Binary	Sex indicates in 1 (woman); 0 (man)
3	Chest pain type	Integer	0typical angina, 1atypical angina, 2non-anginal pain, 3-asymptomatic
4	Resting BPs	Integer	Resting blood pressure in mmHg
5	Cholesterol	Integer	Serum Cholesterol in mm/dL
6	Fasting blood sugar	Binary	Fasting blood sugar levels of a patient
7	Resting ECG	Integer	Resting Electrocardiogram results
8	Max heart rate	Integer	Maximum heart rate achieved in between 60 and 202
9	Exercise angina	Binary	Exercise-induced angina. YYes, NNo.
10	Oldpeak	Integer	Stress test (ST) depression induced by exercise relative to rest
11	ST slope	Integer	The slope of the peak exercise ST segment. Up—upsloping, Flat-flat, Downdownsloping
12	Target	Binary	Working capacity of heart valves and chambers for targeting variable as no disease (0); disease (1)

**Table 3:** Parameters for assessing the performance of our HDP approach.

Parameters	Estimation Formula	Definition
Accuracy	$\frac{TP+TN}{\left(TP+TN+FP+FN\right)}$	Evaluates the method’s ability to accurately discriminate between positive and negative conditions
Precision	$\frac{TP}{\left(TP+FP\right)}$	Percentage of true positives, or correctly identified positive cases, relative to all expected positive cases
Recall	$\frac{TP}{\left(TP+FN\right)}$	Is the proportion of properly detected negatives
AUC (area under curve)	Estimated as the area under the curve.	Symbolizes a collection of data at various ROC (receiver operating characteristic) curve points. The area under the curve (AUC) has a value between 0 and 1

**Table 4:** Different ensemble learning techniques result according to accuracy, recall, precision, and AUC for proposed approach.

Method	Tain Accuracy	Test Accuracy	Recall	Precision	AUC
XGBoost	94.5	91.9	91.7	92	92
LightBoost	95.8	92.16	92.05	92.1	92.05
Voting algorithms	**98.6**	**96.218**	**95.8**	**96.4**	**95.75**

## Data Availability

The original contributions presented in the study are included in the article; further inquiries can be directed to the corresponding author.

## References

[1] Majhi B., Kashyap A. (2024). Explainable AI-Driven Machine Learning for Heart Disease Detection using ECG Signal. Appl. Soft Comput..

[2] Ashraf K., Nawar S., Hosen H., Islam M.T., Uddin M.N. Beyond the Black Box: Employing LIME and SHAP for Transparent Health Predictions with Machine Learning Models. Proceedings of the 2024 International Conference on Advances in Computing, Communication, Electrical, and Smart Systems (iCACCESS).

[3] Ahmed S., Kaiser M.S., Hossain M.S., Andersson K. (2024). A Comparative Analysis of LIME and SHAP Interpreters with Explainable ML-Based Diabetes Predictions. IEEE Access.

[4] Vimbi V., Shaffi N., Mahmud M. (2024). Interpreting artificial intelligence models: A systematic review on the application of LIME and SHAP in Alzheimer’s disease detection. Brain Inform..

[5] Dave D., Naik H., Singhal S., Patel P. (2020). Explainable ai meets healthcare: A study on heart disease dataset. arXiv.

[6] Sethi A., Dharmavaram S., Somasundaram S.K. Explainable Artificial Intelligence (XAI) Approach to Heart Disease Prediction. Proceedings of the 2024 3rd International Conference on Artificial Intelligence for Internet of Things (AIIoT).

[7] Mienye I.D., Jere N. (2024). Optimized Ensemble Learning Approach with Explainable AI for Improved Heart Disease Prediction. Information.

[8] Kavila S.D., Bandaru R., Gali TV M.B., Shafi J. (2022). Analysis of cardiovascular disease prediction using model-agnostic explainable artificial intelligence techniques. Principles and Methods of Explainable Artificial Intelligence in Healthcare.

[9] Varun G., Jagadeeshwaran J., Nithish K., Ds A.S., Venkatesh V., Ashokkumar P. (2024). An Explainable AI Model in Heart Disease Classification using Grey Wolf Optimization. Scalable Comput. Pr. Exp..

[10] Talaat F.M., Elnaggar A.R., Shaban W.M., Shehata M., Elhosseini M. (2024). CardioRiskNet: A Hybrid AI-Based Model for Explainable Risk Prediction and Prognosis in Cardiovascular Disease. Bioengineering.

[11] Divakar C., Harsha R., Radha K., Rao D.V., Madhavi N., Bharadwaj T. Explainable AI for CNN-LSTM Network in PCG-Based Valvular Heart Disease Diagnosis. Proceedings of the 2024 14th International Conference on Cloud Computing, Data Science & Engineering (Confluence).

[12] Tenepalli D., Navamani T.M. (2024). Design and Development of an Efficient Explainable AI Framework for Heart Disease Prediction. Int. J. Adv. Comput. Sci. Appl..

[13] Dharmarathne G., Bogahawaththa M., Rathnayake U., Meddage D. (2024). Integrating explainable machine learning and user-centric model for diagnosing cardiovascular disease: A novel approach. Intell. Syst. Appl..

[14] Akter S.B., Akter S., Sarkar T., Eisenberg D., Fernandez J.F. (2024). Identification of Myocardial Infarction (MI) Probability from Imbalanced Medical Survey Data: An Artificial Neural Network (ANN) with Explainable AI (XAI) Insights. medRxiv.

[15] El-Sofany H.F. (2024). Predicting Heart Diseases Using Machine Learning and Different Data Classification Techniques. IEEE Access.

[16] Kaggle Dataset. https://www.kaggle.com/datasets/sid321axn/heart-statlog-cleveland-hungary-final?select=heart_statlog_cleveland_hungary_final.csv.

[17] Ali P.J., Faraj R.H., Koya E., Ali P.J., Faraj R.H. (2014). Data normalization and standardization: A technical report. Mach. Learn. Tech. Rep..

[18] Singh D., Singh B. (2020). Investigating the impact of data normalization on classification performance. Appl. Soft Comput..

[19] Frazier P.I. (2018). Bayesian optimization. Recent Advances in Optimization and Modeling of Contemporary Problems.

[20] Wang X., Jin Y., Schmitt S., Olhofer M. (2023). Recent Advances in Bayesian Optimization. ACM Comput. Surv..

[21] Kim H., Kim H., Moon H., Ahn H. (2011). A weight-adjusted voting algorithm for ensembles of classifiers. J. Korean Stat. Soc..

[22] Solano E.S., Affonso C.M. (2023). Solar Irradiation Forecasting Using Ensemble Voting Based on Machine Learning Algorithms. Sustainability.

[23] Asselman A., Khaldi M., Aammou S. (2021). Enhancing the prediction of student performance based on the machine learning XGBoost algorithm. Interact. Learn. Environ..

[24] Shyam R., Ayachit S.S., Patil V., Singh A. Competitive Analysis of the Top Gradient Boosting Machine Learning Algorithms. Proceedings of the 2020 2nd International Conference on Advances in Computing, Communication Control and Networking (ICACCCN).

[25] Kumar I.E., Venkatasubramanian S., Scheidegger C., Friedler S. Problems with Shapley-value-based explanations as feature importance measures. Proceedings of the the 37th International Conference on Machine Learning, PMLR.

[26] Speith T. A Review of Taxonomies of Explainable Artificial Intelligence (XAI) Methods. Proceedings of the 2022 ACM Conference on Fairness, Accountability, and Transparency.

[27] Liu J., Mu J., Sun H., Dai C., Ji Z., Ganchev I. (2024). DLGRAFE-Net: A double loss guided residual attention and feature enhancement network for polyp segmentation. PLoS ONE.

